# Global Analysis of DNA Methylation by Methyl-Capture Sequencing Reveals Epigenetic Control of Cisplatin Resistance in Ovarian Cancer Cell

**DOI:** 10.1371/journal.pone.0029450

**Published:** 2011-12-22

**Authors:** Wei Yu, Chengmeng Jin, Xiaoyan Lou, Xu Han, Lisha Li, Yinghua He, Hongyu Zhang, Kelong Ma, Jingde Zhu, Lihua Cheng, Biaoyang Lin

**Affiliations:** 1 Systems Biology Division, Zhejiang–California International Nanosystems Institute (ZCNI), Zhejiang University, Hangzhou, Zhejiang Providence, China; 2 Department of Biology, Technische Universität Darmstadt, Darmstadt Germany; 3 Shanghai Cancer Institute/Renji Hospital, Shanghai Jiaotong Univisity, Shanghai, China; 4 Swedish Neuroscience Institute, Swedish Medical Center, Seattle, Washington, United States of America; 5 Department of Urology, University of Washington, Seattle, Washington, United States of America; UCLA-DOE Institute for Genomics and Proteomics, United States of America

## Abstract

Cisplatin resistance is one of the major reasons leading to the high death rate of ovarian cancer. Methyl-Capture sequencing (MethylCap-seq), which combines precipitation of methylated DNA by recombinant methyl-CpG binding domain of MBD2 protein with NGS, global and unbiased analysis of global DNA methylation patterns. We applied MethylCap-seq to analyze genome-wide DNA methylation profile of cisplatin sensitive ovarian cancer cell line A2780 and its isogenic derivative resistant line A2780CP. We obtained 21,763,035 raw reads for the drug resistant cell line A2780CP and 18,821,061reads for the sensitive cell line A2780. We identified 1224 hyper-methylated and 1216 hypomethylated DMRs (differentially methylated region) in A2780CP compared to A2780. Our MethylCap-seq data on this ovarian cancer cisplatin resistant model provided a good resource for the research community. We also found that A2780CP, compared to A2780, has lower observed to expected methylated CpG ratios, suggesting a lower global CpG methylation in A2780CP cells. Methylation specific PCR and bisulfite sequencing confirmed hypermethylation of PTK6, PRKCE and BCL2L1 in A2780 compared with A2780CP. Furthermore, treatment with the demethylation reagent 5-aza-dC in A2780 cells demethylated the promoters and restored the expression of PTK6, PRKCE and BCL2L1.

## Introduction

Drug resistance is the major reason leading to the high death rate of ovarian cancer. The chemotherapeutic agent cisplatin (cis-diamminedi-chloroplatinum(II)) is particularly effective against ovarian carcinoma with an initial response rate of up to 70% [1]. However, ovarian cancer eventually develops resistant to cisplatin, and the 5-year survival rate for patients is only 15–20% [2]. Cisplatin mediates its actions by forming DNA adducts–primarily intra-strand crosslink adducts [3] and activates several signal transduction pathways include the ATR, p53, p73, and MAPK pathways, resulting in apoptosis [3], [4].

DNA methylation is often associated with transcriptional repression of gene expression [5] and with responses to chemotherapy [6], [7]. One classical example is that methylation of the MGMT (O6-methylguanine-DNA methyltransferase) promoter in gliomas is a useful predictor of the responsiveness of the tumors to alkylating agent carmustine [1,3-bis(2-chloroethyl)-1-nitrosourea], as well as of overall and disease-free survival in gliomas [6]. In ovarian cancers, Taniguchi et al. propose a model for ovarian tumor progression in which the initial methylation of FANCF is followed by FANCF demethylation and ultimately results in cisplatin resistance [7]. DNA methylation of several genes in ovarian cancers including HSulf-1 [8], ABCG2 [9], EZH2 [10] have been found to be associated with drug resistance. Boettcher et al. analyzed high-definition DNA methylation profiles of 800 CpG islands (CGIs) of selected genes and identified that hyper-methylation in CGIs of BRCA1, CDH1, DNAJC15 and SULF2 as well as hypo-methylation of CGIs for ABCB1, APC and HIC1 genes showed increased doxorubicin tolerance [11]. Chang et al. used global gene expression profiling to analyze cancer cells before and after treatment of DNA methyltransferase inhibitor5-aza-2′-deoxycytidine, which re-activates methylation silenced gene [12]. They identified several hundred genes that were down-regulated in cisplatin resistant cancer cells and reactivated by the DNA methyltransferase inhibitor 5-aza-2′-deoxycytidine [12]. Li et al. compared the methylation pattern of A2780 and their derived resistant cell line after several cycles of drug selections using global CGI methylation arrays and mRNA expression microarrays [13].

Taking advantage of the the next-generation sequencing (NGS) technologies, the methylated fraction of genome captured by MeDIP-seq [14], MethylCap-seq [15] and methylcap-seq [16] have been profiled in a greater depth than the array-based platform, revealing many novel regions that are differentially methylated with the biological contents. Here we report the comparison of the global DNA methylation patterns of ciplatin sensitive (A2780) and resistant (A2780CP) ovarian cell lines for the key epigenetic controls responsible for cisplatin resistance. We identified 1224 hyper-methylated and 1216 hypomethylated DMRs (differentially methylated region) in A2780CP compared to A2780. We also found that A2780CP, compared to A2780, has a lower global CpG methylation. Several genes were confirmed to have both promoter methylation and corresponding expression changes including PTK6, PRKCE and BCL2L1, and treatment with the demethylation reagent 5-aza-dC in A2780 cells demethylated the promoters and restored their expression.

## Results

### Methylomic analysis of the cisplatin resistant A2780CP and its isogenic cisplatin sensitive A2780 cell line

**Figure 1:**
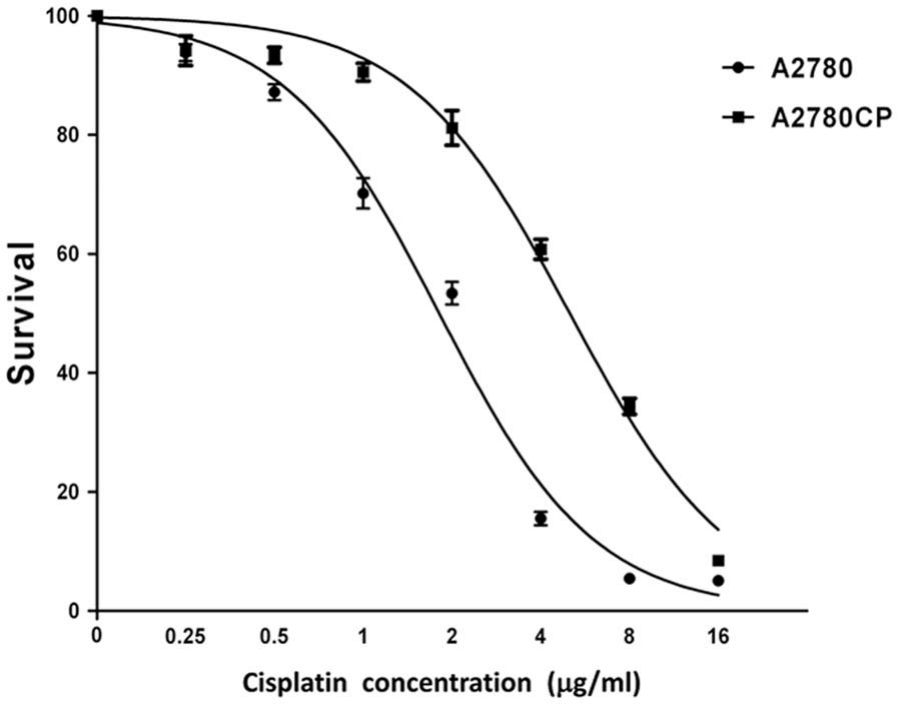
Cell survival curve of A2780 and A2780CP by the MTT assay. Both A2780 and A2780CP were treated with cisplatin in different doses from 0 µg/ml to 16 µg/ml for 72 hours.

Human ovarian carcinoma A2780CP cell line (cisplatin-resistant) was derived from A2780 (cisplatin-sensitive) cell line, but show increased resistance to cisplatin [17]. A2780CP and A2780 are isogenic (same genomic DNA). To confirm that the cell line A2780CP we had still maintained cisplatin resistance, we performed MTT assay to evaluate cisplatin drug responses of A2780CP and A2780 cells. We found that the IC50 of A2780CP (5.0 µg/ml) is nearly 3-fold higher than that of A2780 (1.8 µg/ml) (Fig. 1). Our data is consistent with the previous reported cisplatin response profiles of A2780CP [17], indicating that the cell lines we had in the laboratory retains the difference in cisplatin resistance.

We have therefore applied MethylCap-seq to analyze the methylomic profiles of both A2780 and A2780CP cells. We obtained 21,763,035 raw reads for the drug resistant cell line A2780CP and 18,821,061reads for the sensitive cell line A2780. 13,950,202 (64.1%) and 13,671,899 (72.6%) reads were uniquely mapped to human genome for A2780CP and A2780 respectively.

We annotated the reads with respect to the CpG islands in the human genome (http://genome.ucsc.edu/) and found that about 1.4% and 2.5% of the reads locate inside CpG islands respectively for A2780CP and A2780.The average sequence depth for the CpG islands covered is about 16 and 24.5 times. About 68% and 66% of the human CpG islands were covered in our analysis for A2780CP and A2780 respectively (Table 1). The CpG coverage and depth we obtained in our MethylCap-seq is similar to what previously published [18].

**Table 1:** Coverage of the CpG islands (CGIs) by the MDB-seq and the distribution of DMRs.

Reads in CGIs	Percent in total reads	Mapped CGIs	Percent (mapped CGIs/total CGIs)	Sequence coverage
314,334	1.40%	19,582	68.20%	16
471,279	2.50%	19,215	66.90%	24.5

Hyper-methylated DMRs	DMRs within 5 kb of TSS	DMRs upstream 5 kb of TSS		
1224	633	270		
1216	508	199		

To detect differential methylation regions (DMRs) in the human genome between the sensitive and resistant cells, we used MEDIPS, a recently developed software tool specialized for analyzing immunoprecipitation based methylation analysis (e.g. MeDIP-seq and MethylCap-seq) [19]. We set the criteria for significant DMRs: the length of peaks was set to 500 base pairs and peaks with >20 rpm (reads per million), p-value less than 0.001 and ratio of rpm between two cell line >20. We obtained 1224 DMRs that is hyper-methylated in A2780CP compared to A2780 (Table S1) and 1216 DMRs that is hyper-methylated in A2780 compared to A2780CP (Table S2). These DMRs were annotated with their genomic locations and associated genes within −5 K bp to +5 K bp to the transcription start sites (Table S3 and 4).

**Figure 2:**
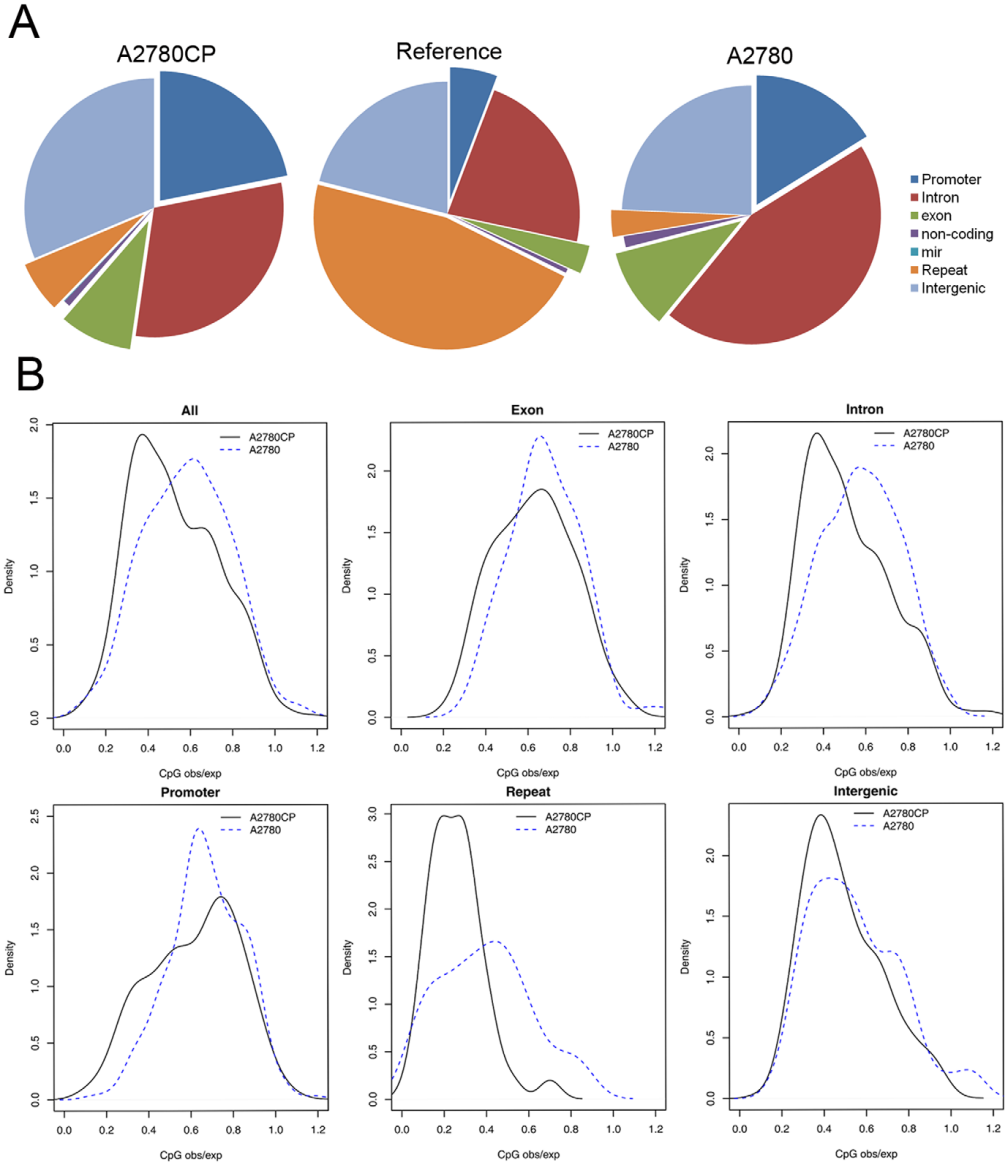
Genomic feature and CpG_o/e_ distribution of the hypermethylated peaks. A. Percentage of hypermethylated peaks based on their localized genomic features. B. Distribution of CpG_o/e_ for hypermethylated peaks based on their localized genomic features.

Nearly half of DMRs were located to within 5 k bp of transcript start sites (TSS) of genes. 190 and 270 DMRs were located to upstream of TSS respectively in A2780 and A2780CP (Table 1). The rest mapped to other regions of the human genome including introns, exons, intergenic regions and repeats (Ensembl human genome annotations) (Fig. 2A). Hypermethylated regions in promoters of genes are known to decrease gene expression [17], but the effect of hypermethylation regions elsewhere within the gene region remains inconclusive. The hypermethylation of repetitive genomic sequences might prevent chromosomal instability, translocations, and gene disruption caused by reactivation of transposable DNA sequences [17], [20].

Li et al. compared isogenic A2780 and their selected resistant cell line using the 60-mer oligo microarray containing 40,000 CpG-rich fragments from 12,000 known gene promoters (Agilent, Santa Clara, CA) [13]. Using a cutoff value of 1.5 fold, they identified 595, 870 and 1,176 hypermethylated genes for the Round1, Round3 and Round5 resistant sublines comparing to the parental (“Round0”) A2780 cells [13]. The technology they used is differential methylation hybridization (DMH) [21], in which methylated DNA was separated from the unmethylated by methylation sensitive enzyme diagestion for probing the oligonucleotide array of the human CpG island regions. DMH is different from MDB-seq, in which methylated DNA was precipitated by recombinant methyl-CpG binding domain of MBD2 protein, and then sequenced. In DMH, isolated DNA was digested with the methylation-insensitive restriction enzyme BfaI (C∧TAG), ligated to linkers, and digested again with the methylation sensitive enzymes HinP1I (G∧CGC) and HpaII (C∧CGG). The digestion products were then amplified using linkers and labeled with Cy3 or Cy5 dyes for comparative hybridizations [21]. Despite different technologies used, we compared our MDB-seq data with Li et al.'s DMH data. We found that 221 (of 1224, 18.1%) and 142 (of 1216, 11.68%) of the hypermethylated and hypomethylated genes in A2780CP in comparison with A2780 were also on the array that Li et al. used (Table S3,S4). We found that 15 regions (corresponding to 13 genes) and 23 regions (21 genes) that are common between the two data sets (Table 2), which are highly significant overlaps with hypergeometric probabilities of 1.11E-5 and 4.22E-20 respectively. The commonly hypermethylated genes in the resistant cells include retinoblastoma binding protein 8 (RBBP8), SRY-box 1 (SOX1), wingless-type MMTV integration site family (WNT9A), general transcription factor IIIA (GTF3), and the commonly hypomethylated genes in the resistant cells include solute carrier family 22 (extraneuronal monoamine transporter) (SLC22A3), aldehyde dehydrogenase 1 family (ALDH1A3), hyaluronan synthase 3 (HAS3) and CUB domain containing protein 1 (CDCP1) (Table 2).

**Table 2:** Common hypermethylated or hypomethylated genes between the MBD-seq and the differential methylation hybridization.

Commonly hypermethylated in resistant cells					
Names	A2780CP*	A2780*	Ratios	Data from Li et al. (GSM385747)**	Descriptions
PROX1	32.86	0.00	Inf	0.98	prospero homeobox 1
PEX5L	24.94	0.00	Inf	0.80	peroxisomal biogenesis factor 5-like
INSM1	41.79	1.26	44.18	0.77	insulinoma-associated 1
JAM3	30.85	0.00	Inf	0.73	junctional adhesion molecule 3
SERF2	31.88	0.00	Inf	0.72	small EDRK-rich factor 2
COL4A1	49.59	2.68	25.51	0.71	collagen
RBBP8	23.06	0.00	Inf	0.70	retinoblastoma binding protein 8
RBBP8	92.64	0.00	Inf	0.70	retinoblastoma binding protein 8
C6orf97	24.91	0.00	Inf	0.69	chromosome 6 open reading frame 97
WNT9A	20.13	0.00	Inf	0.69	wingless-type MMTV integration site family
SEMA6A	33.86	0.50	43.01	0.68	sema domain
GTF3A	28.44	0.00	Inf	0.65	general transcription factor IIIA
SOX1	48.86	2.38	27.04	0.62	SRY (sex determining region Y)-box 1
SOX1	26.26	0.89	38.27	0.62	SRY (sex determining region Y)-box 1
ZNF329	25.72	0.00	Inf	0.62	zinc finger protein 329

Commonly hypermethylated in sensitive cells					
PTMA	0.00	30.98	0.00	−0.95	prothymosin
PTMA	1.29	22.14	0.04	−0.95	prothymosin
SLC22A3	0.00	40.71	0.00	−0.87	solute carrier family 22 (extraneuronal monoamine transporter)
COL18A1	0.00	40.43	0.00	−0.84	collagen
COL18A1	0.00	28.14	0.00	−0.84	collagen
MDFI	0.00	31.38	0.00	−0.83	MyoD family inhibitor
NEFL	0.00	31.08	0.00	−0.83	neurofilament
SECTM1	0.00	26.96	0.00	−0.81	secreted and transmembrane 1
IL20RA	0.00	34.90	0.00	−0.79	interleukin 20 receptor
ADRBK1	0.00	29.51	0.00	−0.78	adrenergic
FBLN1	0.00	28.46	0.00	−0.76	fibulin 1
HSPA12B	0.87	20.44	0.04	−0.76	heat shock 70 kD protein 12B
P2RX5	0.79	23.20	0.05	−0.73	purinergic receptor P2X
LGALS3	0.00	31.78	0.00	−0.71	lectin
ALDH1A3	0.00	22.72	0.00	−0.70	aldehyde dehydrogenase 1 family
SPINT1	0.00	32.23	0.00	−0.69	serine peptidase inhibitor
HAS3	0.17	24.74	0.00	−0.68	hyaluronan synthase 3
TRIP10	0.00	28.15	0.00	−0.65	thyroid hormone receptor interactor 10
CDCP1	0.00	36.70	0.00	−0.64	CUB domain containing protein 1
CDCP1	0.00	20.72	0.00	−0.64	CUB domain containing protein 1
RHOU	0.00	20.43	0.00	−0.61	ras homolog gene family
F3	0.63	22.46	0.03	−0.60	coagulation factor III (thromboplastin)
CXCL14	0.64	24.06	0.02	−0.59	chemokine (C-X-C motif) ligand 14

*rpm, reads per million.

**log2 ratios.

There are 410 hypermethylated DMRs (Table S3) and 316 hypomethylated (Table S4) in A2780CP in comparison with A2780 that were not on the array that Li et al. used, and would not have been identified by the DMH approach. These new DMRs revealed the power of MDB-seq in identifying novel DMRs without prior knowledge of candidate promoters to be put on arrays to be used in DMH. Furthermore, these new DMRs might be due to differences in the derivative resistant cell lines that were used by Li et al. and us.

### A lower global CpG methylation in the cisplatin resistant A2780CP cells compared to the cisplatin sensitive A2780 cells

To understand the global pattern of CpG methylation in both cell lines, we analyzed the characteristics of hypermethylated CpGs across the entire genome by counting the number of neighboring CpGs within the 500 bp regions surrounding hypermethylated sites and calculated the CpG observed/expected ratio (CpG_o/e_) for each CpG using the method described by Ruike et al. [22]. We found that, in general, A2780CP has lower CpG_o/e_ than that of A2780, suggesting a lower global CpG methylation in A2780CP compared to A2780 (Fig. 2B, top left panel). The differences were mostly manifested in the higher CpG_o/e_ in the intron and repeat sequences of A2780 compared to that of A2780CP. Comparing CpG_o/e_ across different genomic region categories (Fig. 2B), the CpG_o/e_ was greater than 0.6 in exons and promoters for both resistant and sensitive cell lines, but less than 0.6 in other genomic regions (repeats, introns and intergenic regions) (Fig. 2B).

### Functional annotations and pathway enrichment analysis for the DMR genes

We performed GO analysis for hypermethylated genes in both the resistant (Table S5) and sensitive cell line (Table S6). We found that hypermethylated genes in the sensitive cell line were enriched for GO terms: anti-apoptosis (GO:0006916), negative regulation of cell death (GO:0060548), and regulation of intracellular protein kinase cascade (GO:0010627). The hypermethylated genes in the resistant cell line have several enriched terms related to transcript factor regulation such as GO:0030528 transcription regulator activity and GO:0003677 DNA binding.

We also performed KEGG pathway analysis for the DRM genes and found that genes with hypermethylated promoters were enriched in the following KEGG pathways including 11 genes in hsa04010 (MAPK signaling pathway), 18 genes in hsa05200 (Pathways in cancer), 5 genes in hsa04310 (Wnt signaling pathway), 5 genes in hsa00982 (Drug metabolism), and 5 genes in hsa04115 (p53 signaling pathway) (Table 3).

**Table 3:** Genes mapped to the enriched pathways.

Pathways (number of genes mapped)	Genes
hsa01100 Metabolic pathways (18)	ACSS1 ALDH1A3 ALDH3A1 B3GNT3 BCAT1 CBS GAD1 GGT7 GLCE GLDC MAN2A2 ME1 PGLS PISD PTGES PTGS1 TPK1 UGT8
hsa05200 Pathways in cancer (18)	BCL2L1 BIRC3 BMP4 CDH1 CDKN1B COL4A1 COL4A2 FGFR1 IGF1R LAMA1 LAMA5 LAMC2 MLH1 RAC2 STAT3 WNT7B WNT9A ZBTB16
hsa04080 Neuroactive ligand-receptor interaction (14)	ADRB2 BDKRB2 CHRNA7 CHRNB4 F2RL1 GABRB3 GLRA3 GRIN2B HRH1 NTSR1 OPRL1 P2RX5 P2RY2 THRB
hsa04514 Cell adhesion molecules (CAMs) (12)	CDH1 CLDN7 CNTNAP2 F11R ICAM1 ICOSLG JAM3 NLGN1 PTPRM PVRL2 SDC2 SIGLEC1
hsa04510 Focal adhesion (12)	BIRC3 CAV1 CCND2 COL2A1 COL4A1 COL4A2 COL6A1 IGF1R LAMA1 LAMA5 LAMC2 RAC2
hsa04010 MAPK signaling pathway (11)	CACNA1G CACNA2D3 CACNG4 DDIT3 DUSP7 FGFR1 GADD45G HSPA1A HSPA1B MAP4K4 RAC2
hsa05145 Toxoplasmosis (10)	BCL2L1 BIRC3 HSPA1A HSPA1B IFNGR2 LAMA1 LAMA5 LAMC2 MYD88 STAT3
hsa04020 Calcium signaling pathway (10)	ADRB2 BDKRB2 CACNA1G CHRNA7 GNA15 GNAL HRH1 NTSR1 P2RX5 6263 RYR3
hsa04512 ECM-receptor interaction (9)	CD44 COL2A1 COL4A1 COL4A2 COL6A1 LAMA1 LAMA5 LAMC2 SDC2
hsa04144 Endocytosis (9)	ADRB2 ADRBK1 CAV1 CHMP6 CXCR4 HSPA1A HSPA1B IGF1R SMAD7
hsa04060 Cytokine-cytokine receptor interaction (9)	ACVR2A CD70 CXCL14 CXCL2 CXCR4 IFNGR2 IL20RA LTBR TNFRSF6B
hsa04974 Protein digestion and absorption (8)	COL15A1 COL18A1 COL2A1 COL4A1 COL4A2 COL6A1 SLC7A8 SLC9A3
hsa05222 Small cell lung cancer (8)	BCL2L1 BIRC3 CDKN1B COL4A1 COL4A2 LAMA1 LAMA5 LAMC2
hsa05146 Amoebiasis (8)	COL2A1 COL4A1 COL4A2 GNA15 GNAL LAMA1 LAMA5 LAMC2
hsa04520 Adherens junction (7)	CDH1 FGFR1 IGF1R PTPRM PVRL2 RAC2 SNAI2
hsa04670 Leukocyte transendothelial migration (7)	CLDN7 CXCR4 F11R ICAM1 JAM3 NCF4 RAC2
hsa04062 Chemokine signaling pathway (7)	ADRBK1 CXCL14 CXCL2 CXCR4 FOXO3 RAC2 STAT3
hsa05322 Systemic lupus erythematosus (7)	GRIN2B H2AFJ HIST1H2BI HIST1H2BM HIST1H3A HIST1H4A HIST1H4I
hsa04630 Jak-STAT signaling pathway (6)	BCL2L1 CCND2 IFNGR2 IL20RA SPRY2 STAT3
hsa04310 Wnt signaling pathway (5)	CCND2 DKK2 RAC2 WNT7B WNT9A
hsa04350 TGF-beta signaling pathway (5)	ACVR2A BMP4 ID4 NOG SMAD7
hsa04360 Axon guidance (5)	CXCR4 EFNA1 EPHA7 RAC2 SEMA6A
hsa00982 Drug metabolism - cytochrome P450 (5)	ALDH1A3 ALDH3A1 GSTA4 GSTT2 MGST2
hsa04650 Natural killer cell mediated cytotoxicity (5)	ICAM1 IFNGR2 RAC2 RAET1L SYK
hsa05142 Chagas disease (American trypanosomiasis) (5)	BDKRB2 GNA15 GNAL IFNGR2 MYD88
hsa00980 Metabolism of xenobiotics by cytochrome P450 (5)	ALDH1A3 ALDH3A1 GSTA4 GSTT2 MGST2
hsa04115 p53 signaling pathway (5)	CCND2 GADD45G PPM1D SERPINB5 SFN

Interestingly, many ECM-related and membrane channel proteins were hypermethylated. For example, KCNS1 and KCNA1, two potassium voltage-gated channel protein, and SLC15A4 (solute carrier family 15, member 4) are hypermethylated in A2780CP cells while SLC4A11 (solute carrier family 4, sodium borate transporter, member 11) is hypermethylated in A2780 cells. COL18A1 (collagen, type XVIII, alpha 1) is hypermethylated in A2780 cells while KRTAP10-6 (keratin associated protein 10–6) and LRFN5 (leucine rich repeat and fibronectin type III domain containing 5) are hypermethylated in A2780CP cells. We also identified hypermethylation at several microRNA loci including hypermethylation of MI6A2, MIR129-2, MIR124-1, MIR124-3, and MIR10A in A2780CP and hypermethylation of MIR185, MIR548Q, MIR642A and MIR661 in A2780 (Table 4).

**Table 4:** Interesting hypermethylated genes and MiRNA loci in A2780 or A2780CP cells.

chr.	start	end	A2780CP*	A2780*	locations	Names	Descriptions
**miRNAs**							
chr12	54383001	54383500	23	0	upstream	MIR196A2	
chr12	54383501	54384000	23	0	upstream	MIR196A2	
chr12	54384501	54385000	26	0	upstream	MIR196A2	
chr12	54387501	54388000	48	1	downstream	MIR196A2	
chr15	96880501	96881000	48	1	downstream	MIR1469	
chr22	22011001	22011500	29	0	downstream	MIR130B	
chr11	43598501	43599000	33	2	upstream	MIR129-2	
chr11	43602501	43603000	30	2	overlapStart	MIR129-2	
chr20	61807001	61807500	33	1	upstream	MIR124-3	
chr20	61807501	61808000	37	0	upstream	MIR124-3	
chr20	61808001	61808500	47	0	upstream	MIR124-3	
chr8	9763501	9764000	22	0	upstream	MIR124-1	
chr8	9762501	9763000	21	1	upstream	MIR124-1	
chr17	46656501	46657000	56	0	downstream	MIR10A	
chr22	20019501	20020000	1	29	upstream	MIR185	
chr10	12763001	12763500	0	20	downstream	MIR548Q	
chr19	46180501	46181000	0	27	downstream	MIR642A	
chr19	46181001	46181500	1	25	downstream	MIR642A	
chr8	145020001	145020500	0	23	upstream	MIR661	
**ECM related genes**							
chr8	122654001	122654500	57	3	upstream	HAS2	hyaluronan synthase 2
chr21	46016001	46016500	26	0	upstream	KRTAP10-6	keratin associated protein 10-6
chr14	42074501	42075000	34	0	upstream	LRFN5	leucine rich repeat and fibronectin type III domain containing 5
chr14	42075001	42075500	33	1	upstream	LRFN5	leucine rich repeat and fibronectin type III domain containing 5
chr14	42075501	42076000	42	2	upstream	LRFN5	leucine rich repeat and fibronectin type III domain containing 5
chr21	46824001	46824500	0	40	upstream	COL18A1	collagen, type XVIII, alpha 1
chr21	46823501	46824000	0	28	upstream	COL18A1	collagen, type XVIII, alpha 1
**membrane channel proteins**							
chr20	43733001	43733500	29	1	upstream	KCNS1	potassium voltage-gated channel, delayed-rectifier, subfamily S, member 1
chr12	5018501	5019000	27	2	upstream	KCNA1	potassium voltage-gated channel, shaker-related subfamily, member 1 (episodic ataxia with myokymia)
chr12	129309001	129309500	26	1	upstream	SLC15A4	solute carrier family 15, member 4
chr20	3220501	3221000	0	50	upstream	SLC4A11	solute carrier family 4, sodium borate transporter, member 11

*rpm, reads per million.

### Validation of genes with DMRs by MS-PCR and bisulfite sequencing

**Figure 3:**
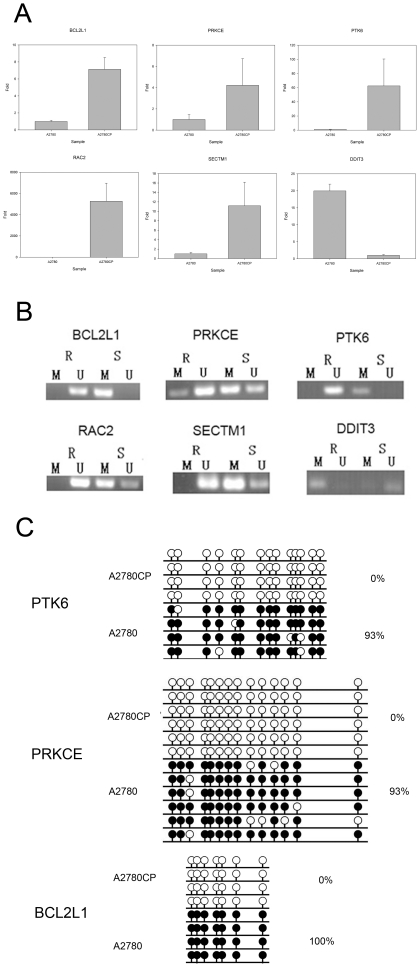
Validation of genes with DMRs in their promoters by MS-PCR and bisulfite sequencing. A. Real-time PCR showing the differential expression of selected genes between the cisplatin sensitive A2780 and the cisplatin resistant A2780CP cells. B. MS-PCR data showing differential methylation states in promoter regions of the selected genes between A2780 and A2780CP cells. C. Bisulfite sequencing result for promoter regions of selected gene PTK6, PRKCE and BCL2L1 (white cycle: unmethylated CpG, Black cycle: methylated CpG).

The expression of genes with hypermethylated promoter was checked by RT-qPCR, if the expression was significant changed then methylation specific PCR (MS-PCR) was used to validate the methylation status of DMR regions between two cell lines. We identified the following genes BCL2L1 (BCL2-like 1), PPKCE (protein kinase C, epsilon), PTK6 (PTK6 protein tyrosine kinase 6), RAC2 (ras-related C3 botulinum toxin substrate 2), SECTM1 (secreted and transmembrane 1) and DDIT3 (DNA-damage-inducible transcript 3) that changed in both promoter methylation and expression. Figure 3A showed the expression changes of these genes, and Figure 3B showed the promoter methylation patterns of these genes. DDIT3 was hypomethylated in A2780 cells compared to A2780CP, while the rest showed the opposite (Fig. 3B). To further confirm DMRs between A2780 and A2780CP, we used bisulfite sequencing to sequence the promoter regions of three randomly picked gene from the 6 genes. Figure 3C showed that all promoter regions of the picked gene PTK6, PRKCE and BCL2L1 were hypomethylated in A2780CP compared with A2780.

### Restoration of gene expression of DMR affected genes by treatmentwith 5-aza-dc demethylation reagent

In order to further confirm our data, we used 5-aza-2′-deoxycytidine (5-aza-dC) to see whether we can re-activate methylation-silenced genes that we identified. 5-aza-2′-deoxycytidine (5-aza-dC) is an analogue of cytosine. When incorporated into DNA, it irreversibly binds the methyltransferase enzymes, resulting in passive de-methylationand reactivation of epigenetically silenced genes [23]. We used 5-aza-dC to treat both A2780CP and A2780 cells in different doses from 0 µM to 10 µM. The expression of genes treated with the lowest (0 µM) was compared to that treated with the highest (10 µM) dose of demethylation reagent.

**Figure 4:**
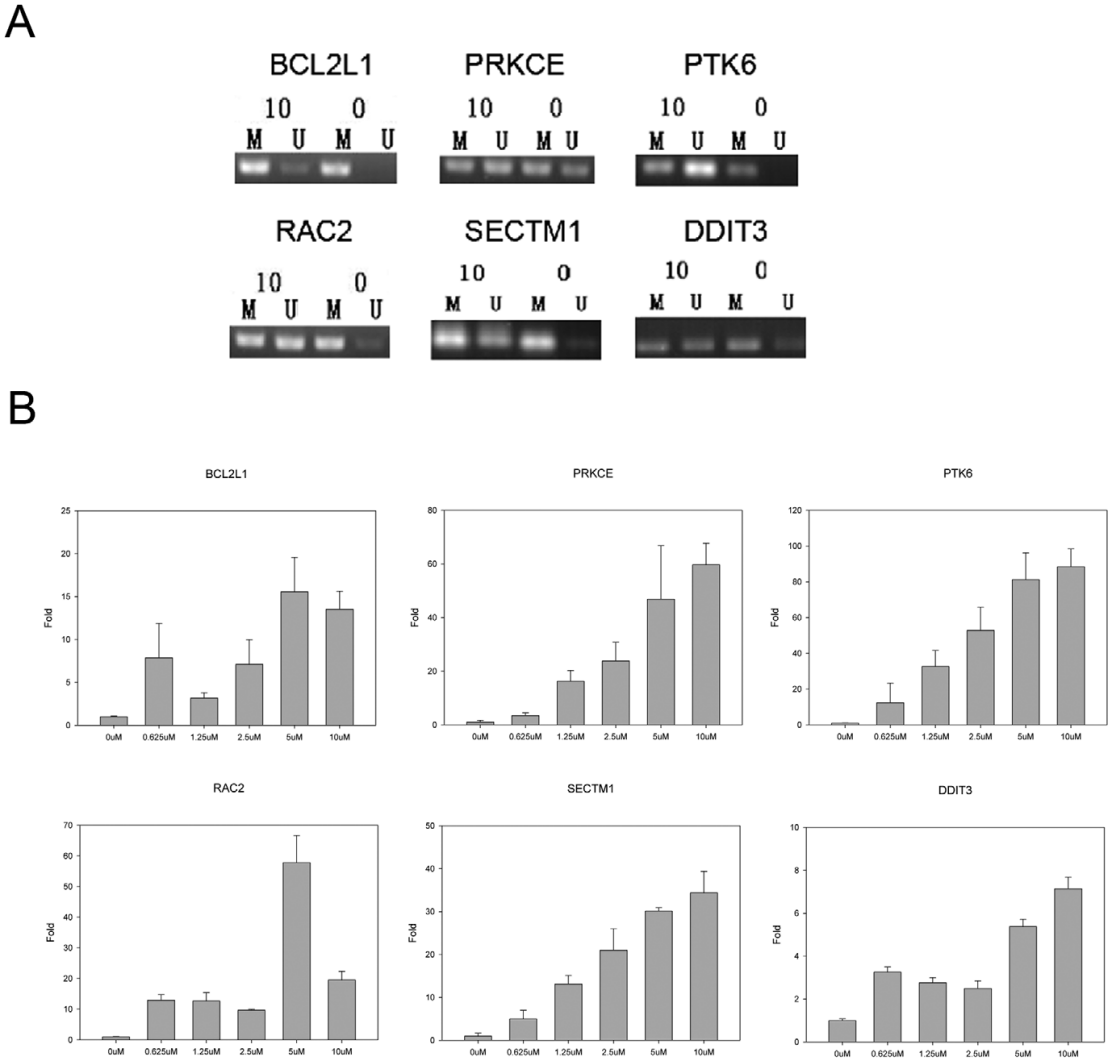
Restoration of gene expression of DMR affected genes by treatment with 5-aza-dc demethylation reagent and validation by MS-PCR. A. Validation of the changes of methylation status by MS-PCR for the six genes under treatment with 0 µM and 10 µM 5-aza-dC. B. Gene expression changes (Y-axis, relative fold changes) of the selected six genes after treatment with different concentration (X-axis) of demethylation reagent 5-aza-dC.

We showed that we were able to demethylate the promoters of the hypermethylated PRKCE, BCL2L1, RAC2, PTK6, SECTM1 (Fig. 4A,B), and restored their expression after treatment with 5-aza-dC in A2780 cells. Similarly, we were able to demethylate the promoters of the hypermethylated gene DDIT3, and restored its expression after treatment with 5-aza-dC in A2780CP cells (Fig. 4A,B).

## Discussion

We describe here for the first time an MDB-seq analysis of a cisplatin response model of ovarian cancer cells. The epigenetic changes between the isogenic pair of the cisplatin sensitive A2780 and its resistant derivative A2780CP cells suggest an epigenetic control mechanism for cisplatin resistance. The global MDB-seq data set generated for this pair of isogenic cells will be a useful resource for the research community interested in cisplatin resistance and epigenetics, and in ovarian cancer in general. Global DNA methylation changes during carcinogenesis and is often a predictor of therapy responses. For example, Anisowicz et al. showed that even the normal part of the lung from a cancer patient has already experienced a loss of global DNA methylation compared to a normal individual [24]. Kim et al. showed that global CpG methylation plays a role in epigenetic control of the radiosensitivity in lung cancer cell lines [25]. In gliomas, LINE-1 methylation, a global DNA methylation marker, is proportional to MGMT promoter methylation and higher LINE-1 methylation is a favorable prognostic factor in primary GBMs, even compared to MGMT promoter methylation [26]. Here we observed a lower global CpG methylation in the cisplatin resistant A2780CP cells compared to the cisplatin sensitive A2780 cells, suggesting that global DNA methylation patterns might be able to predict cisplatin resistance for ovarian cancers. Further experimentation is necessary to evaluate this hypothesis.

We obtained 1,224 and 1,216 DMRs that is hyper-methylated or hypo-methylated in A2780CP compared to A2780 cells (Table S1, S2). We found that hypermethylated genes in the sensitive cell line were enriched for GO terms for anti-apoptosis (GO:0006916) and negative regulation of cell death (GO:0060548), suggesting a possible general mechanism of epigenetic silencing of anti-apoptosis genes, resulting in sensitivity to cisplatin. We also found that the DMRs are enriched in several signaling pathways including the hsa04010 (MAPK signaling pathway), hsa04310 (Wnt signaling pathway), and the hsa04115 (p53 signaling pathway) pathways (Table 3). The Wnt signaling pathway has been implicated in ovarian cancer progression and chemoresistance [27]. Activation of JNK/p38 MAPK pathways in response to cisplatin could lead to Fas ligand induction and cell death in ovarian carcinoma cells [4]. P53 and its signaling pathway were also been implicated in cisplatin resistance in ovarian cancer cells [28], [29]. Our data suggest that epigenetic regulation of these signaling pathways is one of the mechanisms for the involvement of these pathways in cisplatin resistance in ovarian cancer cells.

We also identified hypermethylation at several microRNA loci including hypermethylation of MI6A2, MIR129-2, MIR124-1, MIR124-3, and MIR10A in A2780CP and hypermethylation of MIR185, MIR548Q, MIR642A and MIR661 in A2780 (Table 4). MicroRNAs have been implicated in cisplatin resistance in ovarian cancer [30]. For example, Yang et al. showed that many miRNAs are deregulated in human ovarian cancer including miR-214, miR-199a*, miR-200a, miR-100, miR-125b, and let-7 cluster, and that miR-214 induces cell survival and cisplatin resistance by targeting PTEN [30]. Epigenetically silenced microRNAs have been implicated in cancers [31], [32]. However, methylation of miRNA locus has not been reported previously for ovarian cancers; neither are reports on the relationship between miRNA methylation and cisplatin resistance. Our data might point to a new direction for the study of methylation of microRNA loci and cisplatin resistance.

We found that BCL2L1 (BCL2-like 1), PPKCE (protein kinase C, epsilon), PTK6 (PTK6 protein tyrosine kinase 6), RAC2 (ras-related C3 botulinum toxin substrate 2), SECTM1 (secreted and transmembrane 1) are hypermethylated in the cisplatin sensitive A2780 cells and DDIT3 (DNA-damage-inducible transcript 3) is hypermethylated in the cisplatin resistant A2780CP cells. Their expression also were also changed accordingly to the hypothesis that hypermethylation would silence gene expression. We confirmed the methylation pattern of these genes by MS-PCR, and also were able to demethylate the promoters of these genes and restore their expression after treatment with 5-aza-dC, an agent that de-methylates and reactivates of epigenetically silenced genes [23].

We found BCL2L1 is hypermethylated in the sensitive A2780 cells. BCL2L1 (bcl-xl) encodes a protein belonging to the BCL-2 protein family, whose protein members act as anti- or pro-apoptotic regulators [33]. Over-expression of the Bcl-xL protein is known to confer resistance to a broad range of potentially apoptotic stimuli in carcinogenesis processes including oncogene activation, hypoxia and matrix detachment [34]–[37]. Our result reveals that the hypomethylation of BCL2L1 in the resistant cells (i.e. hypermethylation of BCL2L1 in the sensitive cells) would result in increased BCL2L1 expression, thus conferring resistance to cisplatin. Our data is consistent with the observation by Williams et al. that expression of Bcl-xL in ovarian carcinoma is associated with chemoresistance and recurrent disease [38]. Taking together, this suggest that modulating the expression of BCL2L1 (Bcl-xL) by epigenetic means might be a way to overcome cisplatin resistance in ovarian cancer cells.

We also identified PTK6 (protein tyrosine kinase 6) as hypermethylated in the cisplatin sensitive A2780 cells compared to A2780CP cells. PTK6 directly phosphorylates AKT and promotes AKT activation in response to epidermal growth factor [39]. The relationship of PTK6 with cisplatin has not been previously studied. Further functional analysis of PTK6's role in modulating cisplatin resistance is warranted. PPKCE (protein kinase C, epsilon) is another gene that is hypermethylated in the cisplatin sensitive A2780 cells compared to A2780CP cells. PPKCE is a member of the protein kinase C (PKC) family, whose members phosphorylate a wide variety of protein targets and are involved in diverse cellular signaling pathways [40]. Interestingly, cisplatin was able to induce phosphorylation and translocation of PPKCE from the plasma membrane to the nuclear membrane and to the cytosolic fraction [41]. The hypermethylation of PPKCE in the sensitive cells would reduce its expression, resulting in less translocation to the nuclear membrane or cytosolic fraction upon addition of cisplatin. However, whether reduced expression of PPKCE confers sensitivity to cisplatin in ovarian cancer cells remains to be studied.

Finally, we identified DDIT3 (DNA-damage-inducible transcript 3) as hypermethylated in the cisplatin resistant A2780CP cells compared to A2780 cells. Also named GADD153 (Growth arrest and DNA damage-inducible protein 153), DDIT3 encodes a member of the CCAAT/enhancer-binding protein (C/EBP) family of transcription factors, and is activated by endoplasmic reticulum stress, and promotes apoptosis. DDIT3 (GADD153) expression could be induced by cisplatin [42]. We observed that it is hypermethylation in the resistant cells, which would result in reduced expression of DDIT3. It is possible that cisplatin acts through DDIT3 to promote growth arrest and apoptosis, and reduced expression of DDIT3 in the cells would make them more resistant to cisplatin. However, the detailed mechanism remained to be investigated.

In summary, we generated a global dataset for an ovarian cancer cisplatin resistance model and identified several genes that were subjected to epigenetic control to modulate cisplatin resistance. These genes might serves as targets to overcome chemoresistance to cisplatin in ovarian cancers.

## Methods

### Cell lines

A2780 and A2780CP was cultured in RPMI 1640 medium (Invitrogen) containing 10% Fetal Bovine Serum (10099-141) at 37°C and 5% CO_2_. Cells were kindly provided by Dr. Stephen Collins from UC San Diego (CA, USA).

### Cytotoxicity analysis

Both resistant and sensitive cells were seeded into 96 well plates at a concentration of 4000 cells/well in five replicates with complete culture medium. Then cells were treated with cisplatin in different dose from 0 µg/ml to 16 µg/ml. After 72 hours incubation, both viable and dead cells were counted by using MTT assay, and only the viable cells were included in data analysis.

### Bisulfite-modified DNA sequencing and Methylation-Specific PCR

We prepared genomic DNA from cultured cells using the DNeasy Blood & tissue Kit (Qiagen). Approximately 200 ng of DNA was bisulfite-treated with the EZ DNA Methylation-Gold kit (Zymo Research) according to the manufacture's protocol. ZymoTaq Premix was used for Methylation-Specific PCR (MSP) and according to these primers (Table S7). Methylation-specific PCR was run in a total volume of 25 µL by using ZymoTaq Premix (ZYMO research). MSP reactions were subjected to initial incubation at 95°C for 10 minutes, followed by 40 cycles of 95°C for 30 seconds, and annealing at appropriate temperature for 35 seconds and 72°C for 40 seconds. Final extension was done by incubation at 72°C for 7 minutes. MSP products were separated on 2% agarose gels and visualized by ethidium bromide staining.

The primers for bisulfite-modified sequencing (Table S7) and MSP were designed by web tools MethPrimer (http://www.urogene.org/methprimer/index1.html) [43]. The conditions for bisulfite-modified sequencing reaction are same as MSP. The products were purified using MinElute PCR purification Kit, cloned into the pMD 18-T vector (Takara) and sequenced.

### RNA isolation and Real time RT-PCR

RNA was extracted using the protocol for Trizol reagent (Invitrogen). 2 µg RNA was firstly reverse-transcribed in 25 µL with an Archive Kit (Applied Biosystems) and 2 µL were amplified by Real Time PCR (Bio-RAD CFX96 Rea-Time System). cDNA samples were amplified with SYBR® Pre-mix Ex Taq™. The thermal cycling profile consisted of initial denaturation at 95°C for 30 seconds and 40 cycles at 95°C for 5 seconds, 60°C for 15 second, and 72°C for 30 seconds. Each sample was processed in triplicate.

### 5-Aza-2′-deoxycytidine treatment

Human ovarian carcinoma cells A2780 and A2780CP were grown for 5 days in the presence of various concentrations of 5-Aza-dC (0, 0.625, 1.25, 2.5, 5, and 10 µM). Fresh drug was added every 24 h.

### Methylated DNA Binding Protein sequencing (MethylCap-seq)

3 µg of genomic DNA isolated as described above was fragmented by sonication with Bioruptor (Diagenode, Belgium) and end-repaired, A-tailed, and ligated to 2.5 mMol of “paired-end” adapters (IDT Inc.) following the manufacturer's recommend protocol (Illumina Inc.). 1.2 µg of the DNA was fractionated on a home-made GST-MBD resin with a buffer of a stepwise increased salt concentration [16]. The high salt fraction was directly amplified by 12-cycle PCR [18]. The 350–450 bp fraction of the PCR products was gel purified and quantified using an Agilent DNA 1000.

The 250–400 bp DNA fractions were excised and purified as described above. The products were assessed and quantified using an Agilent DNA 1000 series II assay and Qubitfluorometer (Invitrogen) respectively. Each library was diluted to 8 nM for sequencing on an Illumina Genome Analyzer II following the manufacture's recommended protocol. Obtained images were analyzed and base called using Illumina provided GA pipeline software OLB 1.6.0 with default setting. The raw reads from MethylCap-seq were submitted to GEO database which accession number isGSE31418.

The 250–400 bp DNA fraction of the amplified fraction was gel- purified. Each library was diluted to 8 nM for sequencing by Illumina Genome Analyzer II following the manufacture's recommended protocol. Obtained images were analyzed and base-called using Illumina provided GA pipeline software OLB 1.6.0 with default setting. The raw reads from MethylCap-seq were submitted to GEO database (accession number GSE31418).

### Mapping of Sequence Reads

Human genome sequence and mapping information (Feb. 2009, GRCh37/hg19) was downloaded from ENSEMBL FTP site (http://asia.ensembl.org/info/data/ftp/index.html). Filtered reads were firstly cut into 36 bp to get higher quality reads then mapped to HG19 reference allowing up 2 mismatches by software SOAP2 [44]. The matched results from SOAP2 were converted into bed format by our own script.

### Identification and Annotation of DMRs

To identify differential methylation regions (DMRs), the bed format files were used as input for the MEDIPS program [19]. The criteria for significant DMRs were: the length of peaks was set to 500 base pairs and peaks with >20 rpm (reads per million), p-value less than 0.001 and ratio of rpm between two cell line >20. The length of each DMR was set to 500 bp. Genomic feature data was retrieved from ENSEMBL database (build 55), promoter was defined as the 5 kb upstream of transcription start sites. Each DMR was annotated according to its genomic location by our own script. The DMRs that locate within the proximal promoter (−5 kb to +5 kb from their transcription start site) were annotated with GO, KEGG pathway and Reactome Pathway by package ChIPpeakAnno [45] in R and web tool IDConverter (http://idconverter.bioinfo.cnio.es/) [46].

CpG Obs/Exp ratio was calculated by our own script for each DMR and plotted by the R language. GO Miner [47] was used to analyze GO enrichment and a P value <0.01 was considered significant. Pathway enrichment was analyzed through KEGG database (http://www.genome.jp/kegg/tool/map_pathway1.html) [48]. To compare to Li et al.'s data, we downloaded the data ftp://ftp.ncbi.nih.gov/pub/geo/DATA/SeriesMatrix/GSE15373/. When there are multiple probes corresponding to the same genes, the probe that showed the most differentially expressed were used for comparison. For calculating hypergeometric probabilities, the population size is set at 12,000 as there are 12, 000 genes in the array that Li et al. used.

## Supporting Information

Table S1 **Hypermethylated DMRs in the cisplatin resistant A2780CP cell line.**
(XLS)

Table S2 **Hypermethylated DMRs in the cisplatin sensitive A2780 cell line.**
(XLS)

Table S3 **Hypermethylated DMRs within 5 kb of transcription start sites in the A2780CP cell line.**
(XLS)

Table S4 **Hypermethylated DMRs within 5 kb of transcription start sites in the A2780 cell line.**
(XLS)

Table S5 **Gene Ontology enrichment result for genes with hypermethylated promoters in the A2780CP cell line.**
(XLS)

Table S6 **Gene Ontology enrichment result for genes with hypermethylated promoters in the A2780 cell line.**
(XLS)

Table S7 **Primers for MSP and bisulfite-modified sequencing.**
(XLS)
